# Impact of Reducing Complement Inhibitor Binding on the Immunogenicity of Native *Neisseria meningitidis* Outer Membrane Vesicles

**DOI:** 10.1371/journal.pone.0148840

**Published:** 2016-02-12

**Authors:** Helene Daniels-Treffandier, Karlijn de Nie, Leanne Marsay, Christina Dold, Manish Sadarangani, Arturo Reyes-Sandoval, Paul R. Langford, David Wyllie, Fergal Hill, Andrew J. Pollard, Christine S. Rollier

**Affiliations:** 1 Oxford Vaccine Group, Department of Paediatrics, University of Oxford, and The NIHR Oxford Biomedical Research Centre, Centre for Clinical Vaccinology and Tropical Medicine, Churchill Hospital, Oxford, OX3 7LE, United Kingdom; 2 The Jenner Institute, University of Oxford, Centre for Cellular and Molecular Physiology, The Henry Wellcome Building for Molecular Physiology, Roosevelt Drive, Oxford, OX3 7BN, United Kingdom; 3 Section of Paediatrics, St Mary’s Campus, Imperial College, London, W2 1PG, United Kingdom; 4 Imaxio, 99 rue de Gerland, 69007, Lyon, France; Universidad Nacional de la Plata, ARGENTINA

## Abstract

*Neisseria meningitidis* recruits host human complement inhibitors to its surface to down-regulate complement activation and enhance survival in blood. We have investigated whether such complement inhibitor binding occurs after vaccination with native outer membrane vesicles (nOMVs), and limits immunogenicity of such vaccines. To this end, nOMVs reactogenic lipopolysaccharide was detoxified by deletion of the *lpxl1* gene (nOMV_lpxl1_). nOMVs unable to bind human complement factor H (hfH) were generated by additional deletions of the genes encoding factor H binding protein (fHbp) and neisserial surface protein A (NspA) (nOMV_dis_). Antibody responses elicited in mice with nOMV_dis_ were compared to those elicited with nOMV_lpxl1_ in the presence of hfH. Results demonstrate that the administration of human fH to mice immunized with fHbp containing OMV_lpxl1_ decreased immunogenicity against fHbp (but not against the OMV as a whole). The majority of the OMV-induced bactericidal immune response (OMV_lpxl1_ or OMV_dis)_ was *versus* PorA. Despite a considerable reduction of hfH binding to nOMV_dis_, and the absence of the vaccine antigen fHbp, immunogenicity in mice was not different from nOMV_lpxl1_, in the absence or presence of hfH (serum bactericidal titers of 1:64 *vs* 1:128 after one dose in the nOMV_dis_ and nOMV_lpxl1_–immunized groups respectively). Therefore, partial inhibition of fH binding did not enhance immunity in this model.

## Introduction

*Neisseria meningitidis* is a human-restricted pathogen, which causes meningitis and sepsis, which may result in death or long-term disabilities. The attack rate is highest in young children and adolescents [1]. Based on the carbohydrate composition, thirteen capsular groups of *N*. *meningitidis* have been described. Groups A, B, C, W and Y are the most prevalent worldwide [2] with efficacious protein-polysaccharide conjugate vaccines being available for groups A, C, W and Y. Since polysaccharide-based vaccines against MenB are not efficacious [3], research has focused on sub-capsular antigens for MenB vaccines [4]. Vaccines containing recombinant proteins (used in the two licensed vaccines currently available) and outer membrane vesicles (OMVs) have been used. OMV vaccines have proved especially useful during clonal MenB outbreaks [5, 6].

Native OMVs have potentially higher adjuvant capacity as compared to chemically detoxified OMVs due to the retention of lipopolysaccharide (LPS) [7], but also contain several proteins that bind complement inhibitors including factor H (fH), C4bp and activated vitronectin [8–11]. Binding of such complement inhibitors could potentially reduce the immunogenicity of nOMVs through decreased activation of innate immunity, as alerting and priming the immune system is now a well-recognized function of the complement [12]. A reduction of this signal could result in reduced antigen uptake and presentation by macrophages and decreased production of pro-inflammatory signals. *N*. *meningitidis*, in particular, has the ability to bind human fH (hfH), an inhibitor of the complement alternative pathway (AP) [11, 13]. Three meningococcal surface proteins, factor H binding protein (fHbp), Neisserial surface protein A (NspA) and porin B2 (PorB2), have been implicated in hfH binding [10, 14]. Binding of a host human protein such as fH, transferrin or CECAM1 to a vaccine antigen can interfere with development of the immune response, as demonstrated by studies using human transgenic mouse models [15–17]. There also are supporting data from a non-human primate model [18]. Based on these observations, a nOMV vaccine unable to bind hfH, and thus to inhibit the complement alternative pathway, might elicit a stronger host immune response or have increased adjuvant capacity compared to a wild-type counterpart.

To test this hypothesis, a nOMV vaccine derived from MenB strain H44/76 lacking *fHbp* and *nspA* genes was engineered, and immunogenicity was investigated in mice injected with hfH [19]. This murine model is validated by the fact that treatment of mice suffering from age-related macular degeneration with hfH reverses C3 renal deposition, thus confirming that hfH has functional capacities in mice [19].

## Material And Methods

### Media and Reagents

*Escherichia coli* and *N*. *meningitidis* strains, plasmids and PCR primers used in this study are listed in Table 1. DH5α *Escherichia coli* was grown in Luria-Bertani (LB) broth or on LB-agar plates at 37°C. *N*. *meningitidis* was grown at 37°C in a humidified 5% CO_2_ atmosphere on GC agar plates (Oxoid) supplemented with VitoX (2% v/v, Oxoid SR0090A) or, for genetic manipulations, on Columbia Agar plates supplemented with horse blood (Oxoid) for serum bactericidal assay (SBA). Tryptone Soy Broth (Oxoid) was used for liquid cultures of *N*. *meningitidis*. When required, media were supplemented with antibiotics (Sigma-Aldrich): ampicillin (amp) 100 μg/ml, kanamycin (kan) 50 μg/ml for *E*. *coli* and 100 μg/ml for *N*. *meningitidis*, erythromycin (ery) 300 μg/ml for *E*. *coli* and 5 μg/ml for *N*. *meningitidis*, tetracycline (tet) 5 μg/ml for *E*. *coli* and 2 μg/ml for *N*. *meningitidis*. Normal human serum (NHS) obtained from a healthy adult human volunteer with no previous history of meningococcal disease or immunization was used as complement source for SBA and hfH binding ELISA.

**Table 1:** Bacterial strains, plasmids and primers.

**Bacterial strains:**		**Origin:**
*E*. *coli* DH5α		Laboratory strains collection
*N*. *meningitidis* H44/76-SL		Gift from R. Borrow
*N*. *meningitidis* H44/76 *Δlpxl1*::*tet*		This work
*N*. *meningitidis* H44/76 *ΔnspA *::*kan ΔfHbp *::*ery Δlpxl1*::*tet*		This work
**Plasmids:**		Origin:
pMK-Express		P. Langford (23)
pER2		Van der Voort *et al*., 1996 (24)
pMK-fHbp		This work (GeneArt®)
pMK-nspA		This work (GeneArt®)
pMK-fHbpEry		This work
pMK-nspAKan		This work
**Primers:**	**Sequence (5’-3’)**	**Template**
Kan_forward_PacI	CCGTTAATTAAGGCCGAGCGCAGAAGTGGTC	pMK-Express
Kan_reverse_PacI	CCGTTAATTAATCGGCTCCGTCGATACTATG	pMK-Express
Ery_forward_PacI	GGCTTAATTAACGTTAAACCGTGTGCTCTAC	pER2
Ery_reverse_PacI	CCGTTAATTAACCCTCGAGGTCGACGGTATC	pER2
pMK_forward	TAGGGCGAATTGAAGGAAGG	pMK-GeneArt
pMK_reverse	TGGAAAGCGGGCAGTGAAAG	pMK-GeneArt

### DNA cloning

Chemically competent *E*. *coli* DH5α were prepared using calcium chloride and transformation for the propagation of plasmids was performed using standard methods[20]. Transformed cells were plated onto selective LB agar and incubated at 37°C for 16–18 hours. *N*. *meningitidis* was transformed using the spot transformation technique[21], using 10 μl (10^8^ CFU) of bacterial suspension from an overnight growth and 10 μl of PCR product plated over a 1–2 cm diameter region on GC-VitoX agar. Reactions were incubated at 37°C, 5% CO_2_ for 4–8 hours before bacteria were plated onto selective GC-VitoX agar and incubated for 16–18 hours.

### fHbp::ery, nspA::kan and lpxl1::tet mutations

**Fig 1:**
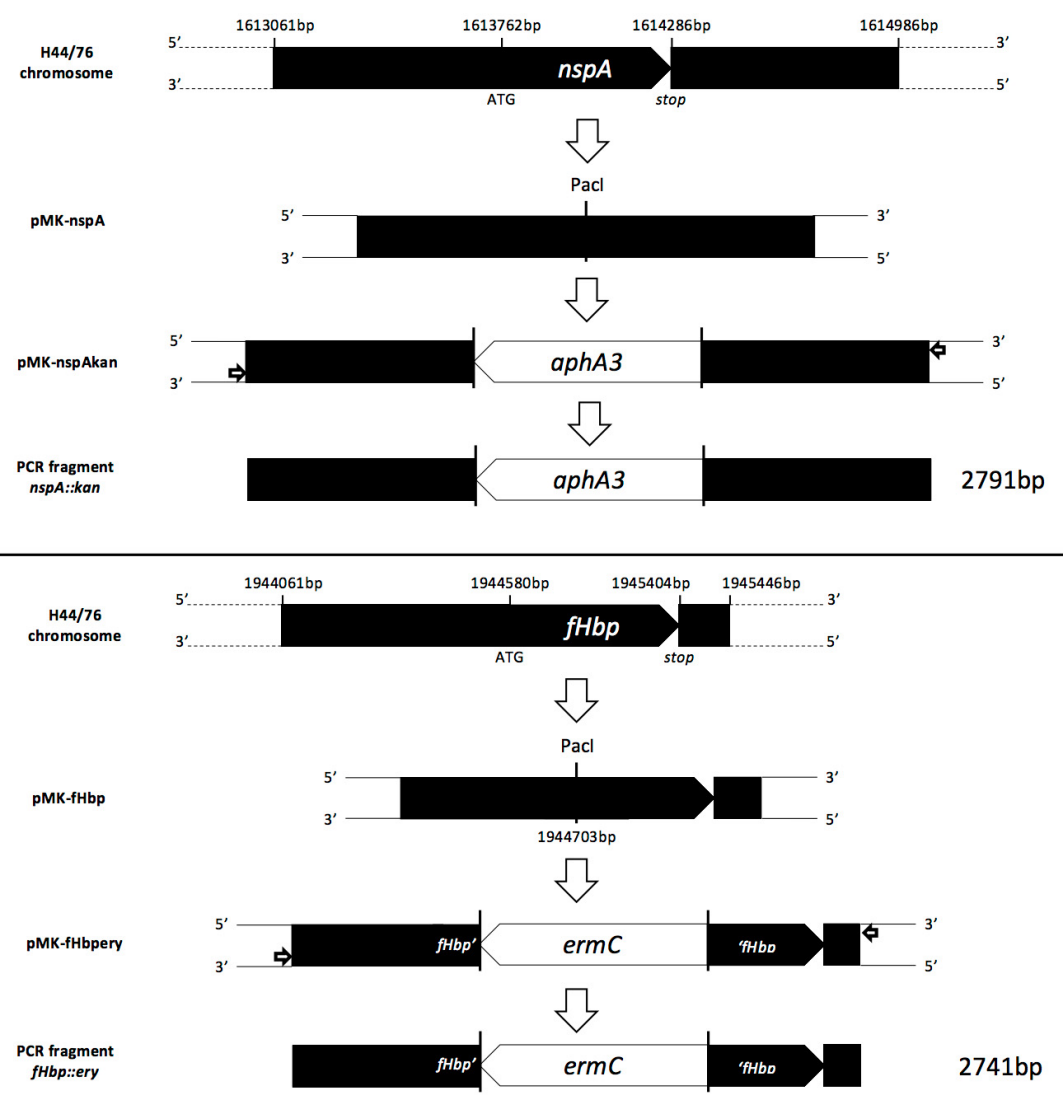
Construction of triple mutant strains. Step1: custom synthesis of plasmids pMK-nspA and pMK-fHbp with a unique PacI restriction site replacing or interrupting *nspA* and *fHbp* ORFs, respectively (GeneArt®, Life technologies). Step 2: counter-clockwise insertion of an antibiotic resistance cassette at the PacI restriction site leading to plasmids pMK-nspAkan and pMK-fHbpery. Step 3: generation of PCR fragments using primers PMK_forward and pMK_reverse.

The *Δlpxl1*::*tet* mutation was introduced in H44/76 as described previously [22] (strain H44/76_Lpxl1_). In parallel, plasmids pMK-nspA and pMK-fHbp were designed with 700bp of both upstream- and downstream- flanking regions of *fHbp* and *nspA*, respectively, with a unique PacI restriction site interrupting or replacing the gene to be deleted. These constructs were synthesised (GeneArt®, Life Technologies). The kanamycin cassette (*aphA3*) with its promoter was amplified by PCR from pMK-Express[23] and the erythromycin cassette (*ermC*) from pER2[24], using primers listed in Table 1. These antibiotics resistance cassettes were inserted at the PacI restriction site into pMK-nspA or pMK-fHbp, respectively, leading to pMK-nspAkan and pMK-fHbpEry. Integration of the cassettes into *N*. *meningitidis* was confirmed by restriction digestion and sequencing. PCRs to amplify the *N*. *meningitidis* DNA region of pMK-nspAkan and pMK-fHbpEry were performed using primers pMK_forward and pMK_reverse (Table 1). Purified DNA fragments (*nspA*::*kan* and *fHbp*::*ery*) were used to transform sequentially H44/76_Lpxl1_ to generate a triple mutant H44/76 *Δlpxl1*::*tet ΔnspA*::*kan ΔfHbp*::*ery* (further referred to as H44/76_dis_) as detailed in Fig 1. At each stage, mutations were checked by PCRs, restriction profiling and confirmed by sequencing.

### Extraction of native OMVs and characterization by SDS-PAGE, Western blotting and ELISA

Native OMVs from H44/76_Lpxl1_ or H44/76_dis_ strains (further referred to as nOMV_Lpxl1_ and nOMV_dis_, respectively) were extracted as previously described[25]: For each strain, bacteria from an overnight culture on GC agar plates supplemented with Vitox (Oxoid) were re-suspended in 2,5ml Tryptone Soy Broth (Oxoid). 100μl of this suspension were plated on 20 fresh GC-Vitox plates and incubated 12 hours at 37°C 5% CO_2_. The totality of the growth was re-suspended in 40ml of OMV buffer (Tris-HCl 0.05M pH7.4, NaCl 0.15M, EDTA 0.01M) and inactivated 40 minutes at 56°C before being sonicated 20x15sec on ice. Cell debris was removed by centrifuging twice for 20 minutes at 16000xg. The supernatant collected was filtered using a 0.22μm filter, before being ultra-centrifuged for 1.5 hours at 250,000xg at 4°C. Finally, the pellet was washed three times with sterile water before being re-suspended in distilled water containing 3% w/v sucrose and stored at 4°C. The expression of fHbp, NspA, PorA, PorB and RmpM was characterized by Western blotting or ELISA as described below. nOMV preparations (6 μg of protein) were boiled for 5min in SDS-PAGE reducing buffer (Bio-Rad, Laemmli 1X #161–0737) before being separated by electrophoresis on a 8–16% Tris-Glycine gel (Biorad, Criterion TGX) and stained with Coomassie blue (SimplyBlue, Invitrogen). For Western blotting; the proteins were transferred to a nitrocellulose membrane (Whatman, Protran 0.2 μm) using a Trans-Blot Turbo transfer system (Biorad). The membrane was blocked over-night using TBST-BSA (TBS, 1%BSA, 0.1% Tween-20) and washed with TBST (TBS, 0.1% Tween-20). The primary antibodies used were: anti-fHbp mAb JAR5 (provided by NIBSC through a gift from Prof. Dan Granoff, Children's Hospital Oakland Research Institute, Oakland CA, USA) diluted 1:5000, anti-PorA p1.7 (NIBSC) diluted 1:5000, anti-PorB p3.15 (NIBSC) diluted 1:750, anti-NspA mAb 236-B2 and anti-RmpM mAb 173,G-1, diluted 1:2 (both gifts from Jan Kolberg, Norwegian Institute of Public Health, Oslo). The secondary antibody was a goat anti-mouse IgG HRP-conjugated pAb (Sigma-Aldrich) diluted 1:50000. Antibody binding was detected with ECL Clarity Western Substrate (Biorad 170–5061) according to the manufacturer’s instructions using a G:box Chemi imager (Syngene). The presence of the major antigen PorA at the surface of both nOMVs was assessed by ELISA: Immulon® 2HB MicrotiterTM plates (Thermo-Scientific) were coated with nOMV_lpxl1_ and nOMV_dis_ at 20 μg/μl at 37°C for an hour. Following washes and a blocking step with PBST-BSA 3% (PBST-BSA), anti-PorA mAb p1.7 (NIBSC) was added to all wells. A goat anti-mouse IgG HRP-conjugate (Jackson Laboratories) was used as the secondary antibody, and 3,3′,5,5′-Tetramethylbenzidine liquid substrate (TMB, Sigma-Aldrich) used for detection according to the manufacturer’s instructions.

### Evaluation of the binding of human fH to nOMVs

96-wells plates were coated with different concentrations of nOMVs (5 μg/ml, 10 μg/ml or 20 μg/ml) overnight at 4°C in the absence of detergent. After washing with PBST (PBS1X, Tween® 20 0.05%), plates were blocked in PBST-BSA 3%. Normal human serum (NHS) was heated at 56°C for 30 min to inactivate the complement, thus preventing C3b deposition, which could serve as an alternative ligand for hfH, on bacteria. This treatment is known not to affect hfH binding properties to fHbp [26]. The wells were washed and incubated with different concentrations of de-complemented NHS (100%, 50%, 25% or none) or with purified hfH (Complement Technologies) for 10 mins at 37°C. Primary and secondary antibodies were diluted in TBST-BSA3% and incubations were performed at room temperature for 1 hour. The antibodies used were: mouse anti-hfH mAb (AbD Serotec MCA508G) diluted 1:250 and goat anti-mouse IgG (Jackson Laboratories) diluted 1:25000. Antibody binding was determined as described above.

### Mouse immunizations

All procedures were performed in accordance with the terms of the UK Home Office Animals Act Project License. Procedures were approved by the University of Oxford Animal Care and Ethical Review Committee. Samples were obtained following terminal general anaesthesia. In all experiments, groups of five to eight 6-week old female C57bl/6 mice (Harlan) were immunized intramuscularly with nOMV_Lpxl1_ or nOMV_dis_, at day 0 and 28. Vaccines contained 5.1 mg/ml Al(OH)_3_ as an adjuvant, according to the manufacturer’s instructions (Brenntag Biosector, Denmark). Mice were immunized at day 0 and 28 with alum-adsorbed vaccines at two different concentrations (2.5 or 5 μg of proteins). Blood was collected under terminal general anaesthesia two weeks after the second dose (day 42). To investigate the effect of the absence of hfH binding to nOMV_dis_, nOMVs (5 μg) were pre-incubated for 1 hour with 800 μg/ml hfH or 800 μg/ml hSA (Human Serum Albumin, Sigma-Aldrich) as an irrelevant control protein, in phosphate buffered saline pH 7.2. To mimic the presence of hfH *in vivo* in mice[19], groups of C57bl/6 mice were injected intraperitoneally with 500 μg hfH or 500 μg hSA 24 hours and 30 mins prior to immunization with 5 μg non-adjuvanted nOMV_dis_ or nOMV_lpxl1_ at day 0. Two control groups consisted of non-immunized mice injected with or without 500μg hfH. Levels of hfH in mice serum were assessed by ELISA on 50μl of blood collected by tail bleed at various time points (30 mins, 24 hours, 48 hours and 72 hours) after the second hfH injection. Blood was collected 2 weeks post immunization. Sera were separated by centrifugation and stored frozen at -20°Cuntil further analysis.

### Evaluation of hfH levels and antibody responses in murine serum

hfH levels in mice were evaluated by sandwich ELISA. Briefly, Immulon® 2HB plates were coated for one hour at 37°C with sheep anti-hfH antibody (AbD Serotec #4400–9504). Mouse sera were added to the plates and incubated for an hour at room temperature under gentle rocking. Purified hfH was used as a standard (1 mg/ml, Complement Technology Inc, #A137). Mouse anti-hfH mAb (AdB Serotec MCA508G) was added to the plates prior to the secondary goat anti-mouse IgG HRP-conjugated antibody (Jackson Laboratories), and the reaction was revealed as described above. For antigen-specific antibody responses, whole cell, recombinant PorA and fHbp ELISA were performed as described previously [22]. IgG titres were calculated as the reciprocal of the last dilution giving an optical density readout superior to the cut-off (four time the average O.D. obtained with naïve sera).

### Serum Bactericidal Assay (SBA)

Human complement-mediated SBA titres were measured in pooled or individual sera when these were available in sufficient quantities, using wild-type 44/76-SL as the target strain (gift from Prof R. Borrow, PHE North West Laboratory, Manchester). The complement source was obtained from healthy donors who had provided written informed consent (Ethics number 10/H0102/23). The donation trial was conducted in accordance with the clinical trial protocol and the principles of the Declaration of Helsinki (2008) and the International Conference on Harmonization (ICH) Good Clinical Practices standards. The analyses were performed in microtiter plates by the standardized method [27]. CFU were counted using an automated counter (Sorcerer; Perceptive Instruments, Haverhill, Suffolk, United Kingdom). The bactericidal titers were defined as the reciprocal of the serum dilution that killed at least 50% of the organisms.

### Statistical analysis

Calculations were carried out using GraphPad PRISM version 6.00 for Mac (GraphPad Software, San Diego California, USA, www.graphpad.com). A one-way analysis of variance (ANOVA) was conducted on the log_10_ transformed antibody titers data to test for differences between groups. If the data appeared to be non-normal, non-parametric Kruskal Wallis analysis was carried out with Dunn’s adjusted p-values for multiple comparisons.

## Results

### Characterization of nOMVs

**Fig 2:**
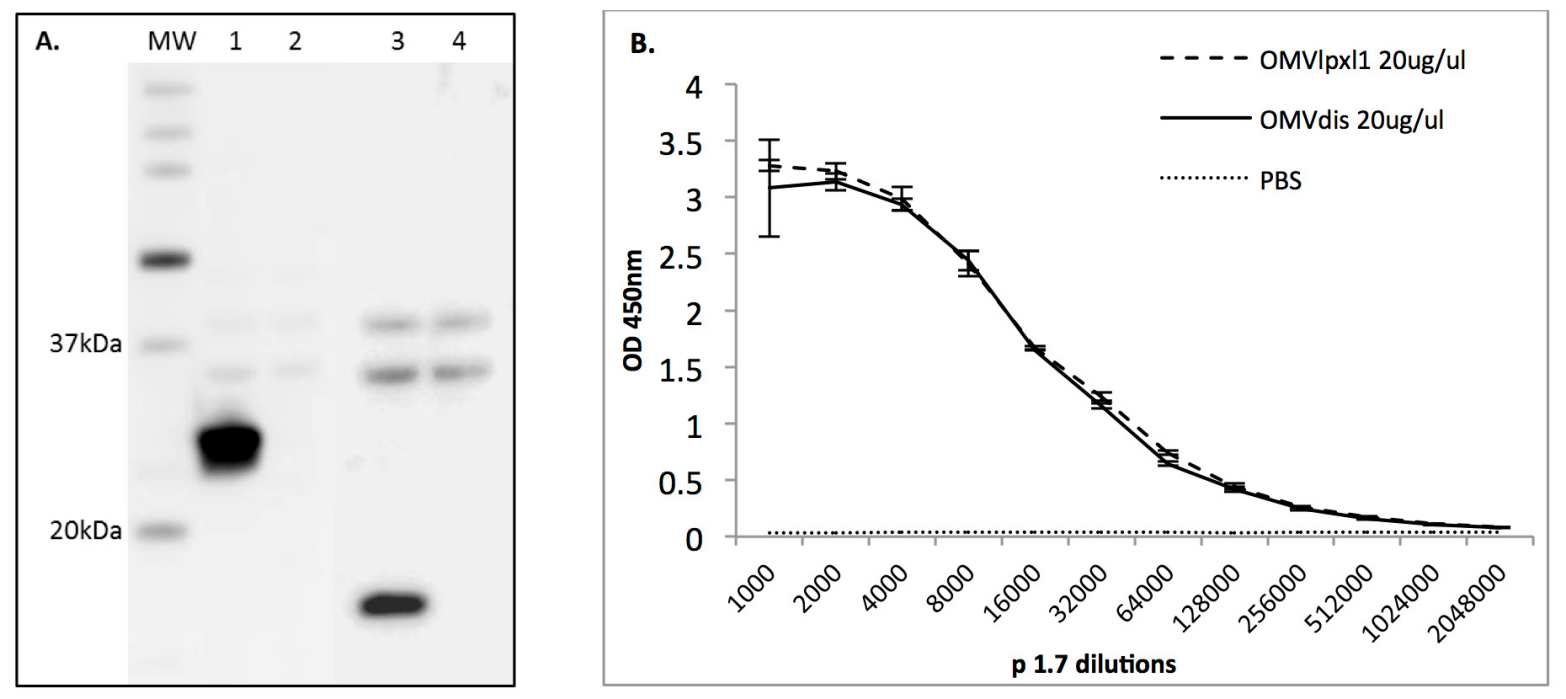
Characterization of nOMVs. **A.** Expression of fHbp and NspA in the nOMVs. nOMV_lpxl1_ (lanes 1 and 3) and nOMV_dis_ (lanes 2 and 4) proteins were separated by SDS-PAGE followed by separate western blotting against fHbp (lane 1 and 2), or NspA (lane 3 and 4). Molecular weight of protein standard (MW) is indicated. **B.** Expression of PorA in the nOMVs. The relative amount of PorA in nOMV_dis_ (dash-line) was compared to nOMV_lpxl1_ (plain line) by ELISA with α-PorA mAb (p1.7). Data presented are the means ± S.D. of two independent assays, each performed in duplicate.

The strains H44/76 *Δlpxl1*::tet and H44/76 *Δlpxl1*::*tet ΔnspA*::*kan ΔfHbp*::*ery* were confirmed by PCR and restriction digestion profiling. nOMV_lpxl1_ and nOMV_dis_ were produced from H44/76 *Δlpxl1*::tet and H44/76 *Δlpxl1*::*tet ΔnspA*::*kan ΔfHbp*::*ery*, respectively. Evaluation of protein concentrations showed similar yields between preparations (7.25 μg/μl for nOMV_lpxl1_ against 4.85 μg/μl for nOMV_dis_). As expected, *ΔfHbp*::*ery* and *ΔnspA*::*kan* mutations led to a total suppression of fHbp and NspA protein expression in nOMV_dis_ compared to nOMV_lpxl1_ (Fig 2A). These mutations did not impact on PorA expression, a major surface antigen of *N. meningitidis [28],* as shown by ELISA (Fig 2B), nor that of other antigens such as PorB and RmpM (data not shown).

### Absence of fHbp and NspA reduces hfH binding to nOMVs

**Fig 3:**
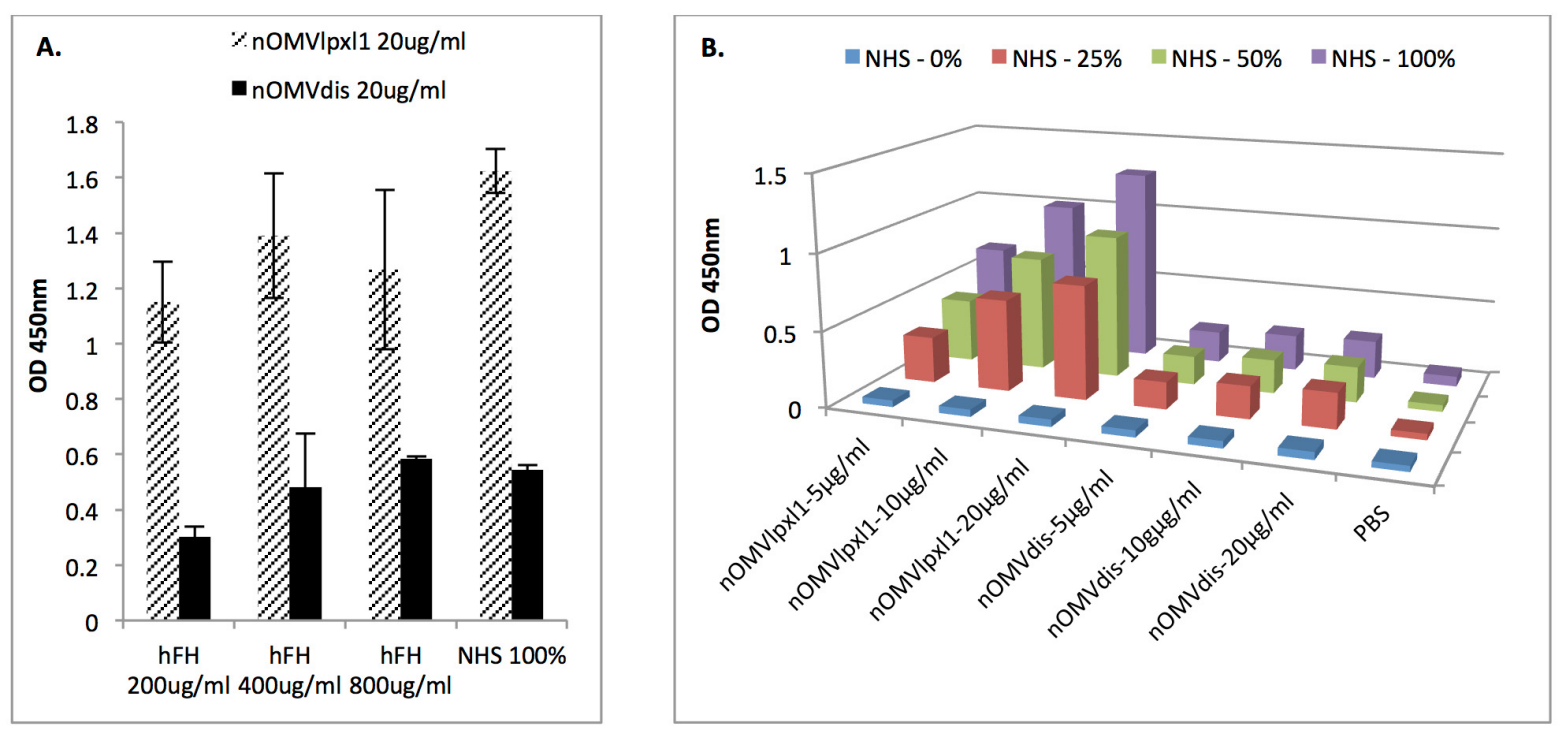
hfH binding to nOMV_dis_ as compared to nOMV_lpxl1_. **A.** Binding of purified hfH at different concentrations (200, 400 and 800 μg/ml) or of de-complemented NHS to 20 μg/ml of nOMV_lpxl1_ (hashed bars) and nOMV_dis_ (dark bars). Means ± S.D. of two independent assays, each performed in duplicate, are presented. **B.** Detection of hfH binding from NHS on nOMVs at three different concentrations of nOMVs (5, 10 or 20 μg/ml) and three different concentrations of de-complemented NHS (25, 50 or 100%).

While nOMV_Lpxl1_ bound at similar levels to the three concentrations of purified hfH tested (with ODs of 1.15, 1.39 and 1.27 for the 200, 400 and 800 μg/ml hfH concentrations, respectively), binding of purified hfH to nOMV_dis_ was reduced, with the OD being on average 35.6% less as compared with that of nOMV_lpxl1_ (OD 0.30 to 0.58, Fig 3A). When NHS was used as a source of hfH, a dose–response was observed with nOMV_lpxl1_, with ODs ranging from 0.30 to 1.27. By contrast, the ODs observed with nOMV_dis_ ranged from 0.18 to 0.25 (Fig 3B). Thus, absence of fHbp and NspA substantially reduced hfH binding to nOMV_dis_.

### Absence of fHbp and NspA did not impair nOMVs immunogenicity in mice

**Fig 4:**
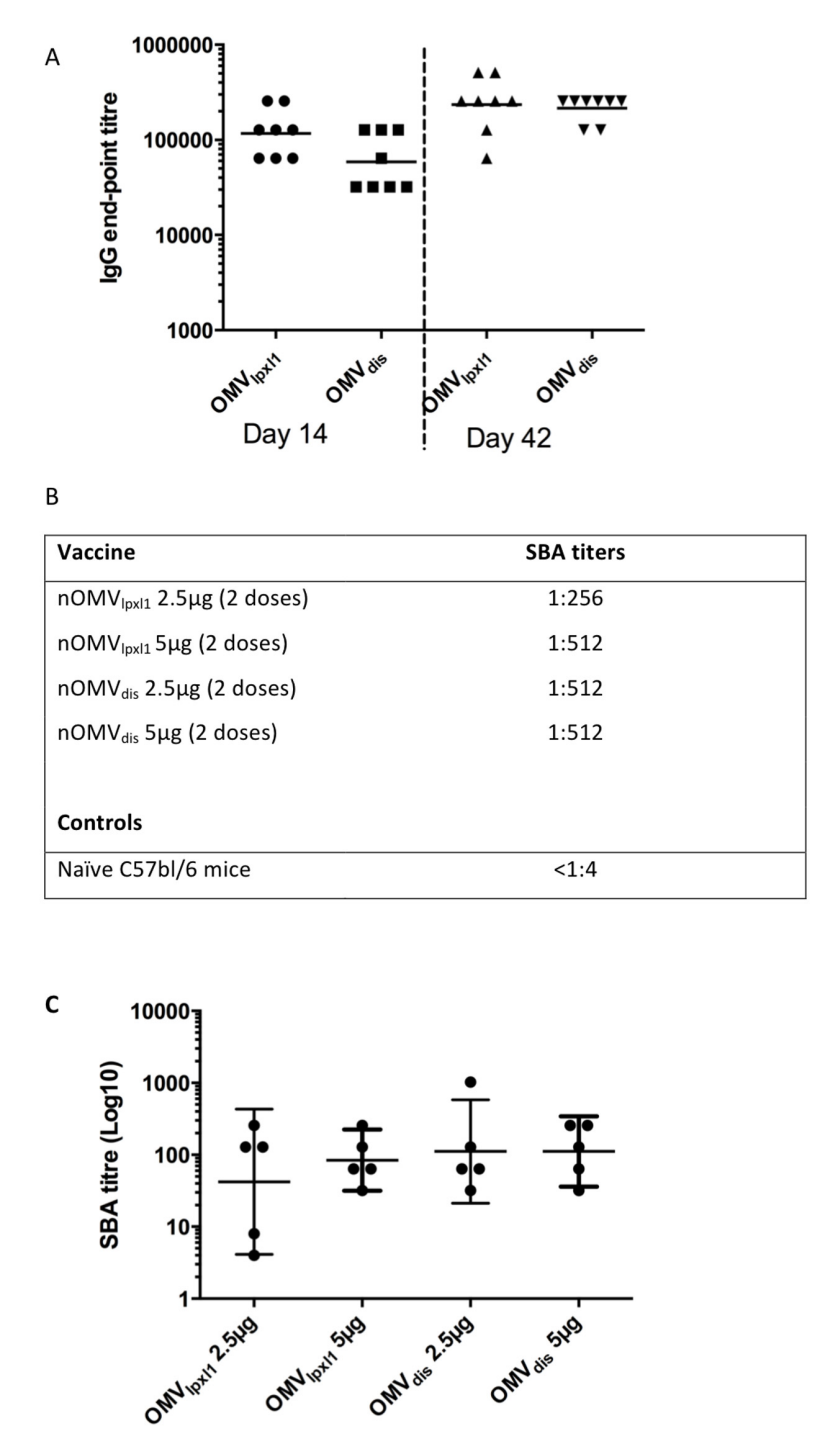
nOMVs immunogenicity in C57Bl/6 mice. **A.** Whole cell-specific IgG titres in C57/bl6 mice sera following immunization with 5 μg nOMV_lpxl1_ or nOMV_dis_. Data represent the individual antibody titers in each animal, and the geometric mean of the group, at day 14 (2 weeks post first injection) and day 42 (2 weeks post second injection). These results are representative of 2 independent mouse experiments. There was no statistical difference between the two groups at day 14 (p = 0.085) nor at day 42 (p = 0.069) by two-tailed Mann-Whitney test. **B.** SBA titers against 44/76-SL wild-type in C57bl/6 mice pooled sera at day 42 (14 days following booster immunization) with 2,5μg or 5μg of nOMV_lpxl1_ or nOMV_dis_. **C**. Individual SBA titers against 44/76-SL at day 42, dots represent individual animals, the geometric mean and 95% confidence interval are represented as horizontal bars.

fHbp is a major surface-exposed antigen of *N*. *meningitidis*, being highly expressed on strain H44/76 [29, 30], and it is one of the most promising vaccine candidates against capsular group B *N. meningitidis [31]*. Therefore, the deletion of this antigen could lead to a loss in immunogenicity of nOMV_dis_ compared to nOMV_lpxl1_ in wild-type mice. As shown in Fig 4A, however, C57BL/6 mice immunized with nOMV_dis_ produced similar amounts of H44/76 whole cell- specific IgG as compared to mice immunized with nOMV_lpxl1_ after the first and second injections. The pooled bactericidal antibody titers were also similar, with titers of 1:256 and 1:512 induced by 2.5 and 5 μg nOMV_lpxl1_, respectively and 1:512 with nOMV_dis_ (Fig 4B). Individual SBA analysis at day 42 suggested that the SBA in pooled sera is an over-estimation of the average response in individual mice, but confirmed that no difference between groups was detectable (Fig 4C). Thus absence of NspA and fHbp did not impair nor increase the immunogenicity of nOMV_dis_ suggesting that these 2 proteins have a limited contribution to the nOMV-induced antibody response in this model.

### Antibody responses in mice in the presence of hfH

**Fig 5:**
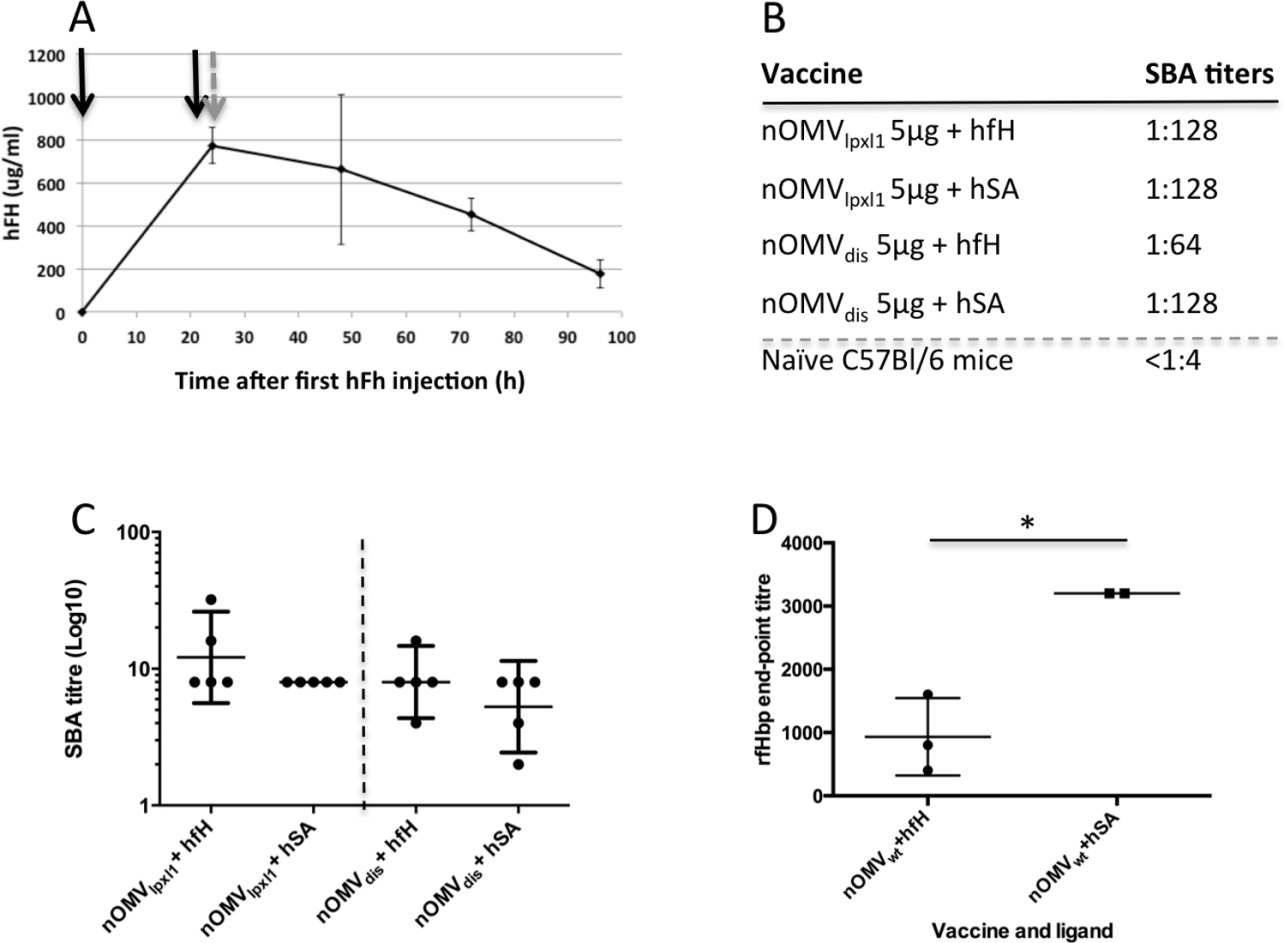
Effect of the presence of hfH in mice on the nOMV immunogenicity. **A.** Evolution of hfH concentration in mouse serum over time after two IP injection of 0.5mg purified hfH (black arrows) 24 and 0.5 hours prior to immunization (grey arrow). **B.** Serum Bactericidal Assay (SBA) titers against 44/76-SL wild-type in pooled sera from C57bl/6 mice pre-injected with 800 μg/ml hfH or hSA, 14 days following immunization with 5 μg of nOMV_lpxl1_ or nOMV_dis_. **C**. Individual SBA titers against 44/76-SL, dots represent individual animals **D**. Individual ELISA titers against rfHbp induced after one dose of H44/76_Lpxl1_ OMV in mice pre-injected with hfH or hSA, *p = 0.016, not all sera were available. **C, D** dots represent individual animals, the geometric mean and 95% confidence interval are represented as horizontal bars.

Mice were injected intraperitoneally with hfH prior to vaccination to reach the standard human serum concentration (500 μg– 800 μg/ml). While hfH was not detectable in mice prior to hfH injection, the concentration of hfH in mouse serum reached around 750 μg/ml after two IP injections of 500 μg hfH 24 hours apart (Fig 5A). This concentration is close to the maximum of the human range of hfH concentration (500–800 μg/ml). However, the bactericidal titer elicited by one vaccination with nOMV_dis_ in mice pre-injected with hfH (1:64) was not significantly different from that obtained with nOMV_lpxl1_ (1:128, Fig 5B). It was also similar for both nOMV preparations in control mice pre-injected with human serum albumin (1:128, Fig 5B), demonstrating that in this model, deletion of *fHbp* and *nspA* had no impact on the nOMV immunogenicity. Individual SBA titers were lower than the SBA titer obtained with the pooled serum, but confirmed that no difference was observed between the groups (Fig 5C). To confirm that the administered hfH induced the expected decreased antibody response against fHbp, individual fHbp-specific antibody responses of mice injected with human fH or the control human serum albumin (hSA) and immunized with H44/76_Lpxl1_ OMV (containing fHbp) were measured in sera that were available. Although not all sera were available, the ELISA titers induced against rfHbp after a single dose H44/76_Lpxl1_ OMV were lower in the group that received hfH passive administration as compared to the control (Fig 5D, p = 0.016, unpaired t test). Of note, no booster dose was administered to avoid the confounding effect of raising anti-hfH antibodies in mice. Bactericidal assays were performed against a PorA off strain to investigate whether the antibody response against other minor (non-PorA) antigens are affected by deleting *fHbp* and *nspA*. However SBA titers induced by one injection of nOMV against a PorA off strain were very low or undetectable, and no statistically significant difference were detected between groups (data not shown).

### Antibody response in mice following immunization with nOMV_lpxl1_ or nOMV_dis_ pre-incubated with purified hfH

**Fig 6:**
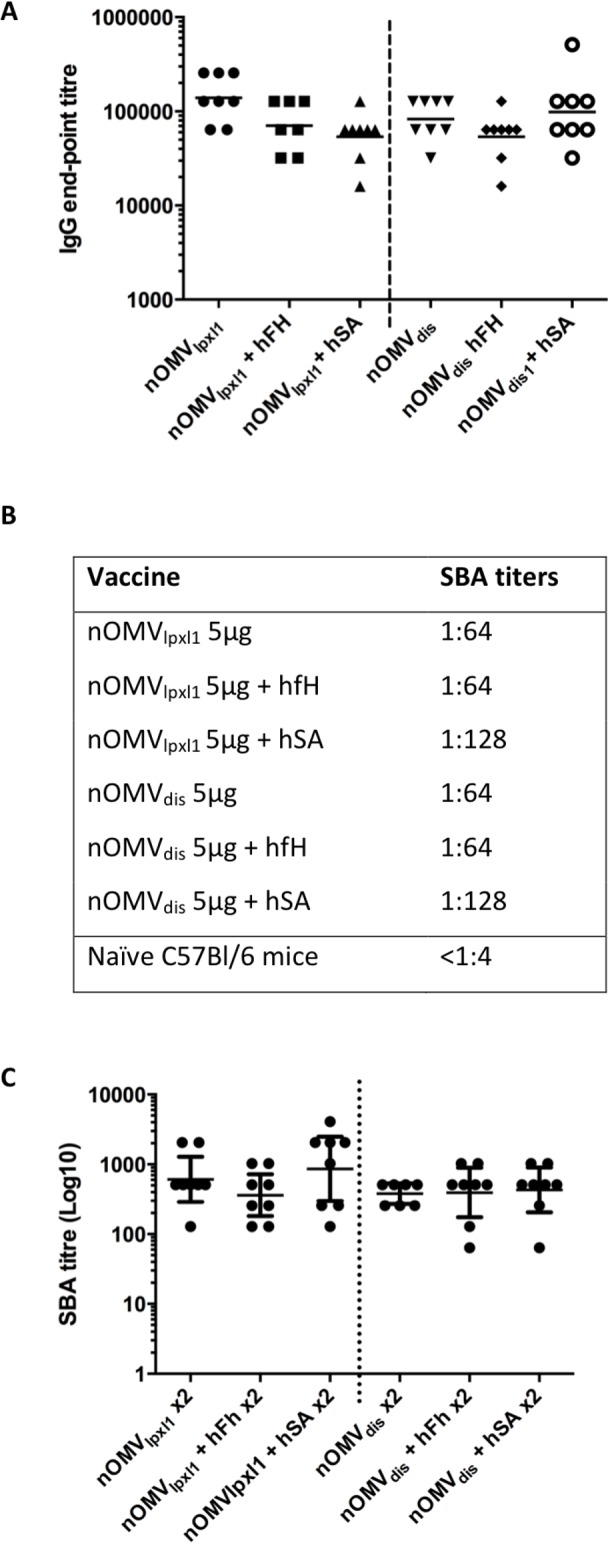
Immunogenicity of nOMVs pre-incubated with purified hfH. Specific IgG response in C57/bl6 mice sera following immunization with 5 μg nOMV_lpxl1_ and nOMV_dis_ pre-incubated or not with 800 μg/ml hfH or hSA, assessed by ELISA using heat-inactivated H44/76 whole cells. Data represent the individual antibody titers in each animal, and the geometric mean of the group, at days 14 (**A)** (2 weeks post single injection) There were no statistical differences between the 6 groups according to statistical analysis performed on log-transformed data using Kruskal-Wallis test with Dunn’s multiple comparison correction. **B.** SBA titers against 44/76-SL in C57bl/6 mice pooled sera at day 14. C. Individual SBA titers at day 42 (2 weeks post second immunization), dots represent individual animals, the geometric mean and 95% confidence interval are represented as horizontal bars.

In the previous experiment, intraperitoneal injections led to short-lived levels of hfH in mice, and raised the possibility that there was not enough hfH in the appropriate compartment at the time of priming the immune response. Therefore, a model of *ex vivo* pre-incubation of the vaccines (nOMV_lpxl1_ or nOMV_dis_) with hfH at 800 μg/ml was established. In this experiment, the vaccines were not adjuvanted with aluminum hydroxide to avoid any putative inhibition of the binding between nOMVs and hfH by alum. To avoid the confounding factor of raising anti-hfH in mice, only a single injection was performed. The nOMV-specific IgG response in mice immunized with nOMV_dis_ pre-incubated with hfH was similar to the controls *i*.*e*. nOMV_dis_ incubated with an irrelevant (hSA) or without hfH pre-incubation at day 14 (Fig 6A). The same level of IgG production was observed in mice immunized with nOMV_lpxl1_ alone or pre-incubated with hfH or hSA, suggesting that the reduced binding of hfH to the nOMV_dis_ did not have any quantitative effect on the antibody response. Similarly, the SBA against 44/76-SL (Fig 6B) did not show any significant difference (more than a titer) between the six vaccinated groups (titers of 1:64–1:128), suggesting that reduced binding of hfH to nOMV_dis_ did not enhance the immune response. At day 28, a booster dose was administered after pre-incubation as described above, and individual SBA titers analyzed two weeks later (Fig 6C). Results confirmed that no difference was observed between groups. SBA titers against a PorA off strain were also measured to investigate whether the antibody response against other minor (non-PorA) antigens was modified, but SBA titers against a PorA off strain were very low or undetectable, and there were no statistically significant differences between groups (data not shown).

## Discussion

The insertion of mutations in *N*. *meningitidis* H44/76 led to a total suppression of fHbp and NspA expression in nOMV_dis_, while the amount of PorA remained unaffected. In the triple mutant H44/76_dis_ there was a reduction in hfH binding of around 35%, consistent with previous observations with **Δ***fHbp***Δ***nspA* H44/76 mutants [32]. However, despite the reduction of human fH binding in H44/76_dis_, immunogenicity of derived nOMV_dis_ in mice was not significantly different from nOMV_lpxl1_ in the presence of hfH, either injected into mice or directly pre-incubated with the nOMVs prior to injection.

In this study, hfH binding to nOMV_dis_ was reduced but not abrogated. Lewis and collaborators obtained similar results with a capsular group B *N*. *meningitidis* strain in which *fHbp* and *nspA* were deleted and the LPS unsialylated [32], and showed that PorB2 is implicated in the inhibition of the complement alternative pathway by interacting with hfH [33]. However H44/76 does not express PorB2. So far, other meningococcal components have been implicated in hfH sequestration such as PorB3 in certain strains [34]. Our work suggests the existence of one (or several) other meningococcal proteins able to inhibit complement alternative pathway by sequestering hfH and highlights the multiplicity and complexity of the strategies capsular group B *N*. *meningitidis* has evolved to evade the human complement cascade. The hfH binding may not have been sufficiently suppressed by deleting *fHbp* and *nspA*, and the residual binding thus makes it difficult to interpret the importance of partially reduced hfH binding on overall immunogenicity. Of note, *N*. *meningitidis* is capable of binding the serum complement inhibitor C4bp [9] and activated vitronectin, which could also impact on immunogenicity.

We had hypothesized that nOMV_dis_ would induce higher complement activation compared to hfH binding nOMVs, which in turn would result in an increased adaptive response to the nOMVs [12], as previously observed with fHbp antigen [35]. In a previous study, increased antibody response was observed against an OMV containing a point-mutated fHbp unable to bind hfH, as compared to an OMV containing a wild type fHbp able to bind hfH [36]. Therefore, the increased immunogenicity was observed against the hfH ligand (fHbp), in contrast to our study where fHbp and NspA are absent and the remaining hfH ligand on the disarmed OMV is unknown. Direct binding of the complement inhibitor to the antigen may be necessary for complement inactivation to impact on the antigen’s resulting immunogenicity. An example of this phenomenon was shown in human CEACAM transgenic mice, only the responses to the ligand for human CEACAM, Opa, was decreased, but not the responses to the OMV as whole [17].

The relevance of the murine model could be questioned since complement evasion by *N*. *meningitidis* by complement inhibitor sequestration is specific to humans. A hypothesis could be that mice lack other factors implicated in *N*. *meningitidis* resistance to complement. However, treatment of mice suffering from age-related macular degeneration with hfH reverses C3 renal deposition[19], thus suggesting that hfH has functional capacities in mice. In addition, our data suggest, despite the low number of mice, that the passive administration of hfH decreased the antibody response to fHbp in mice immunized with H44/76_Lpxl1_ OMV, in agreement with previous published results [15, 36]. Transgenic mice expressing full length human fH [15], or a chimeric fH consisting of mouse fH CCPs 1–5 and 9–20 (enabling interaction with murine C3b and other complement components), flanking hfH CCPs 6–8 (allowing binding to fHbp) as previously described [37] may have been better predictor of the effect in human, but these models were not available for testing. Our results suggest that the effect of human fH on the immunogenicity of fHbp occurs at a step soon following immunization. When human fH is injected, the human fH is present only for a few days at best (serum hfH concentration peaks 24hours after injection, and is down to very low levels at 96 hours[19]), unlike the human fH transgenic mice, yet its effect on fHbp immunogenicity is noted. This provides a mechanistic insight: it is likely that antigen presentation and processing is affected by fH binding to fHbp, as opposed to further downstream processes.

We observed that nOMVs lacking fHbp are as immunogenic as wild-type nOMVs. In the absence of fHbp and NspA, both nOMV-specific IgG responses and SBA titers in mice are similar to nOMVs extracted from the wild-type strain, which is in agreement with previous observations that SBA responses induced by OMVs are largely directed against PorA[38]. Depletion of antigens such as fHbp could unmask sub-dominant epitopes, and support a re-direction of the immune response towards other antigens. Creating a triple Δ*fHbp*, Δ*nspA*, Δ*porA* mutant would allow the assessment of whether the absence of these major antigens reveals sub-dominant antigens or cryptic epitopes. Moreover, PorA also binds the complement inhibitor C4bp, allowing *N*. *meningitidis* to escape the classical- and lectin complement pathways [9]. Disrupting hfH binding as well as C4bp binding to *N*. *meningitidis* might ameliorate the adjuvant properties of nOMVs extracted from such a strain, as OMVs have been considered for their carrier and adjuvant properties[5], in particular, for *Haemophilus influenzae type b* and malaria vaccines candidates [39, 40].
